# Altered fecal bile acid composition in active ulcerative colitis

**DOI:** 10.1186/s12944-023-01971-4

**Published:** 2023-11-18

**Authors:** Stefanie Sommersberger, Stefan Gunawan, Tanja Elger, Tanja Fererberger, Johanna Loibl, Muriel Huss, Arne Kandulski, Sabrina Krautbauer, Martina Müller, Gerhard Liebisch, Christa Buechler, Hauke Christian Tews

**Affiliations:** 1 Department of Internal Medicine I, Gastroenterology, Hepatology, Endocrinology, Rheumatology and Infectious Diseases, University Hospital Regensburg, 93053 Regensburg, Germany; 2 Institute of Clinical Chemistry and Laboratory Medicine, University Hospital Regensburg, 93053 Regensburg, Germany

**Keywords:** Fecal calprotectin, Cholic acid, Deoxycholic acid, Ulcerative Colitis, Crohn´s Disease, Bile acids

## Abstract

**Background:**

Disturbed bile acid homeostasis associated with a rise of primary and a decline of secondary bile acids is a consistent finding in inflammatory bowel diseases (IBDs). Whether fecal bile acids may emerge as biomarkers for IBD diagnosis and disease severity is less clear. Our study aimed to identify associations of 18 fecal bile acid species with IBD entity and disease activity.

**Methods:**

Stool samples of 62 IBD patients and 17 controls were collected. Eighteen fecal bile acid species were quantified by LC–MS/MS using stable isotope dilution. Lipid levels normalized to a dry weight of the fecal homogenates and ratios of single bile acid species to total bile acid levels were used for calculations.

**Results:**

IBD patients exhibited altered primary and secondary bile acid ratios in stool, with notable distinctions between ulcerative colitis (UC) compared to Crohn’s disease (CD) and healthy controls. Fecal calprotectin was negatively correlated with glycolithocholic acid (GLCA) and hyodeoxycholic acid (HDCA) in UC. These bile acids were reduced in stool of UC patients with fecal calprotectin levels > 500 µg/g compared to UC patients with low calprotectin levels. Moreover, negative associations of six secondary bile acids with C-reactive protein (CRP) existed in UC. In CD patients, fecal bile acids did not correlate with CRP or fecal calprotectin. Diarrhoea is common in IBD, and UC patients with diarrhoea had reduced deoxycholic acid (DCA), glycine conjugated DCA (GDCA) and lithocholic acid in stool in contrast to patients with normal stool consistency. Fecal bile acid levels were not associated with diarrhoea in CD patients. UC patients treated with mesalazine had increased levels of fecal GDCA whereas no such changes were observed in CD patients. Bile acid levels of CD and UC patients treated with biologicals or corticosteroids did not change. Relative levels of GHDCA (specificity: 79%, sensitivity: 67%) and glycochenodeoxycholic acid (specificity: 74%, sensitivity: 63%) were the most specific to distinguish UC from CD.

**Conclusion:**

Disrupted fecal bile acid homeostasis is associated with disease severity and disease symptoms in UC but not in CD, potentially aiding in distinguishing IBD subtypes and classifying the pathophysiology of diarrhoea in UC.

**Supplementary Information:**

The online version contains supplementary material available at 10.1186/s12944-023-01971-4.

## Background

Bile acid synthesis in the liver and secretion into the intestine is the major route for the elimination of cholesterol. Hepatocytes produce the primary bile acids cholic acid (CA) and chenodeoxycholic acid (CDCA), which are conjugated with taurine or glycine before their release into the bile fluid [1, 2]. Bile acids, which are not reabsorbed in the ileum, are metabolized by gut microbiota. This involves deconjugation of primary bile acids and their transformation into secondary bile acids with deoxycholic acid (DCA) and lithocholic acid (LCA) being the most prevalent forms. The secondary bile acid ursodeoxycholic acid (UDCA) is derived from CDCA through C7beta epimerization [1, 2]. Hyodeoxycholic acid (HDCA) is produced from LCA by gut bacteria [3] and, while most bile acid species in serum of patients with inflammatory bowel disease (IBD) were decreased, HDCA serum levels were increased [4].

IBDs with ulcerative colitis (UC) and Crohn´s disease (CD) as main entities are characterized by mucosal inflammation of the gastrointestinal tract [5–7]. Convergent evidence supports dysbiosis of the gut microbiota in IBD, which is characterized by a reduction in microbial diversity [2, 8–10]. Consequently, fecal bile acid composition of IBD patients is modified. Most studies agree that fecal levels of primary bile acids of IBD patients are increased, and levels of secondary bile acids are decreased in comparison with healthy controls [9, 11].

The deconjugation of glycoursodeoxycholic acid (GUDCA) to UDCA, the transformation of CA to DCA, and the desulfation of LCA-3 S to LCA are impaired in IBD patients, particularly during inflammatory flare-ups of the disease [11]. It must be noted that there are also studies which failed to identify major differences of fecal bile acid levels between IBD patients and healthy controls [12, 13]. There are considerable inter-individual variabilities of bile acid levels [14, 15], and this may hamper the identification of IBD specific patterns.

Most bile acids are reabsorbed via transport proteins in the distal ileum. Apical sodium dependent bile acid transporter (ASBT) expression was reduced in ileal biopsies taken from patients with CD compared with healthy controls [16, 17]. Inflammatory cytokines contribute to low ASBT levels in IBD, and steroids, which exert anti-inflammatory activities and can induce remission of IBD, may restore ASBT expression [17–19]. Intestinal bacteria can degrade budesonide and mesalazine, another anti-inflammatory compound for IBD therapy [20]. Evidence suggests that drugs for IBD therapy change the microbiome composition, and may improve bile acid malabsorption [20].

Excess bile acids within the colon can cause diarrhoea, and diarrhoea is a common symptom of IBD [21]. The pathogenesis of diarrhoea in IBD is still unclear, and according to previous studies, diarrhoea in IBD patients is not caused by excessive fecal loss of bile salts [21, 22]. In IBD, diarrhoea is secondary to gut inflammation and conditions such as malabsorption of carbohydrates and dietary fat [23]. This is why the magnitude of diarrhoea does not necessarily reflect the severity of IBD [18, 23].

The association of fecal bile acid levels with IBD disease activity is still controversial. Total concentrations of fecal secondary bile acids or levels of conjugated bile acids could not distinguish active disease from remission in IBD [11]. DCA levels in stool of CD patients responding to anti-TNF therapy did not differ from non-responders [24].

On the other hand, correlations of fecal bile acid species with laboratory markers of systemic inflammation of UC patients have been reported. This study showed positive associations of fecal primary bile acids and negative correlations of fecal secondary bile acids with blood markers of inflammation [25]. In this study, C-reactive protein (CRP), as the most used systemic inflammatory marker, was not included [25–27].

Fecal calprotectin is the most important non-invasive biomarker for IBD disease activity in daily clinical practice and correlates with endoscopic activity [28, 29]. As far as we know, associations of fecal bile acids with fecal calprotectin of IBD patients (as a marker for mucosal disease activity) have not been reported. Whether fecal bile acids may emerge as biomarkers of IBD disease activity thus needs further investigation [2, 30]. Our study aimed to comprehensively analyze fecal bile acid species to identify those related to clinical markers of inflammation and disease severity, enhancing understanding of disease pathologies.

## Materials and methods

### Patients

Patients with IBD were randomly assigned from the outpatient or inpatient department of our tertiary centre (Department of Internal Medicine I, University Hospital Regensburg). Patients were recruited for the study from December 6, 2021, to January 31, 2023. IBD was diagnosed based on histologic, endoscopic and clinical criteria [31]. Patients who had coagulopathy were not included in the study.

### Stool collection and analysis of fecal bile acids

Feces from patients as well as healthy controls (hospital staff, students and partners of the patients) were collected at participants’ homes in 70% isopropanol. Upon arrival of these samples at the hospital, feces were stored at -80 °C until use.

Stool samples were homogenized in a gentleMACS^™^ Dissociator (Miltenyi Biotec GmbH, Bergisch Gladbach, Germany). The dry weight of the homogenate was determined by drying 1.0 ml of the mixture in a vacuum centrifuge. For further analysis, the raw feces homogenates were diluted to a final concentration of 2.0 mg dry weight/ml. Fecal bile acids were quantified by LC–MS/MS with a modified method for serum using stable isotope dilution analysis [32, 33].

The primary bile acids cholic acid (CA), its glycine and taurine conjugates (GCA and TCA, respectively) as well as chenodeoxycholic acid (CDCA) and its conjugated forms GCDCA and TCDCA were measured. The analyzed secondary bile acids included deoxycholic acid (DCA), lithocholic acid (LCA), ursodeoxycholic acid (UDCA) and hyodeoxycholic acid (HDCA), and the corresponding glycine and taurine conjugated forms.

### Statistical analysis

Data are shown as boxplots or bar charts. The bars show the mean values ± SEM. Circles or asterisks in the boxplots mark outliers. Mann Whitney U-test, Kruskal-Wallis Test, Receiver Operating Curve (ROC) analysis and Spearman correlation were the statistical tests used (SPSS Statistics 26.0 program, IBM, Leibniz Rechenzentrum, München, Germany). Data in tables are listed as median, minimum, and maximum values. Data were corrected for multiple comparisons and a value of *P* < 0.05 was considered significant.

## Results

### Fecal bile acid levels of IBD patients in comparison with healthy controls

The study cohort consisted of 17 healthy controls and 62 patients with IBD (CD *n* = 38, UC *n* = 24). Patients with CD and patients with UC showed comparable levels of C-reactive protein (CRP) and fecal calprotectin (Table 1). Details of the study cohorts are summarized in Table 1.

**Table 1:** Characteristics of the study groups. There were no significant differences between these groups (Body mass index: BMI; Glomerular filtration rate: GFR)

Characteristics	IBD	CD	UC	Controls
Number (female/male)	62 (28 / 34)	38 (20 / 18)	24 (8 / 16)	17 (10 / 7)
Age (years)	42 (19–78)	42 (19–70)	39 (20–65)	48 (23–78)
BMI (kg/m^2^)	24 (16–44)	24 (17–44)	25 (16–43)	not determined
C-reactive protein (mg/L)	3 (0–144)	4 (0–44)	2 (0–144)	not determined
Creatinine (mg/dL)	0.85 (0.51–1. 25)	0.80 (0.59–1.25)	0.87 (0.51–1.12)	not determined
GFR (mL/min)	99 (61–136)	100 (61–131)	98 (62–136)	not determined
Fecal calprotectin (µg/g)	62 (17–1616)	60 (17–1527)	73 (19–1616)	not determined

The levels of the 18 bile acid species analyzed were similar in feces of females and males in the control cohort and the IBD patients. CA (r = 0.691, *P* = 0.013) and CDCA (r = 0.660, *P* = 0.023) positively correlated with age in the control group. Fecal bile acids did not correlate with the age of the patients (*P* > 0.05 for all).

**Fig. 1:**
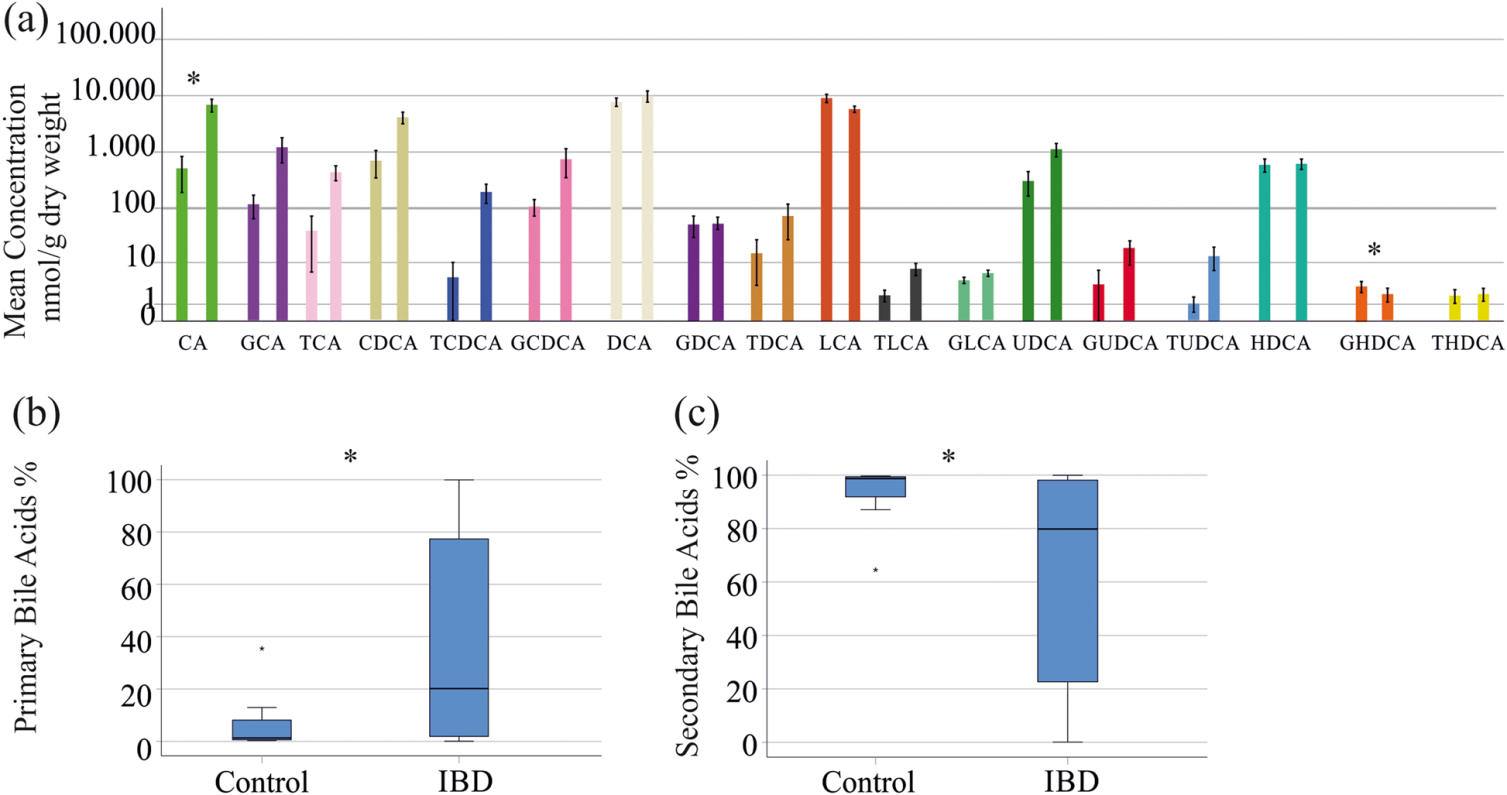
Comparison of fecal bile acid levels between patients with IBD and controls. **a** Concentrations of bile acid species in stool of healthy controls (left bars) and IBD patients (right bars). Data are shown in a logarithmic scale to improve the visualization of low abundant bile acid species; **b** Levels of primary bile acids relative to total bile acid concentrations of IBD patients and controls; **c** Levels of secondary bile acids relative to total bile acid concentrations of IBD patients and controls. * *P* < 0.05

In comparison between IBD patients and healthy controls, we identified lower levels of the glycine conjugated bile acid HDCA (GHDCA, *P* = 0.018) in stool of IBD patients and higher levels of CA (*P* = 0.047) (Fig. 1a and Table S1).

Bile acid levels showed high inter-individual variations (Fig. 1a), and therefore, % of single bile acid species relative to total bile acid levels were calculated. Here, %CA (*P* = 0.040) was higher and %LCA (*P* = 0.045) was lower in IBD. Accordingly, the proportion of primary bile acid levels relative to total bile acid concentrations was increased (*P* = 0.01) and that of the secondary bile acids relative to total bile acid concentrations of IBD patients was decreased (*P* = 0.01) in comparison to healthy controls (Fig. 1b, c).

### Fecal bile acids of CD and UC patients

**Fig. 2:**
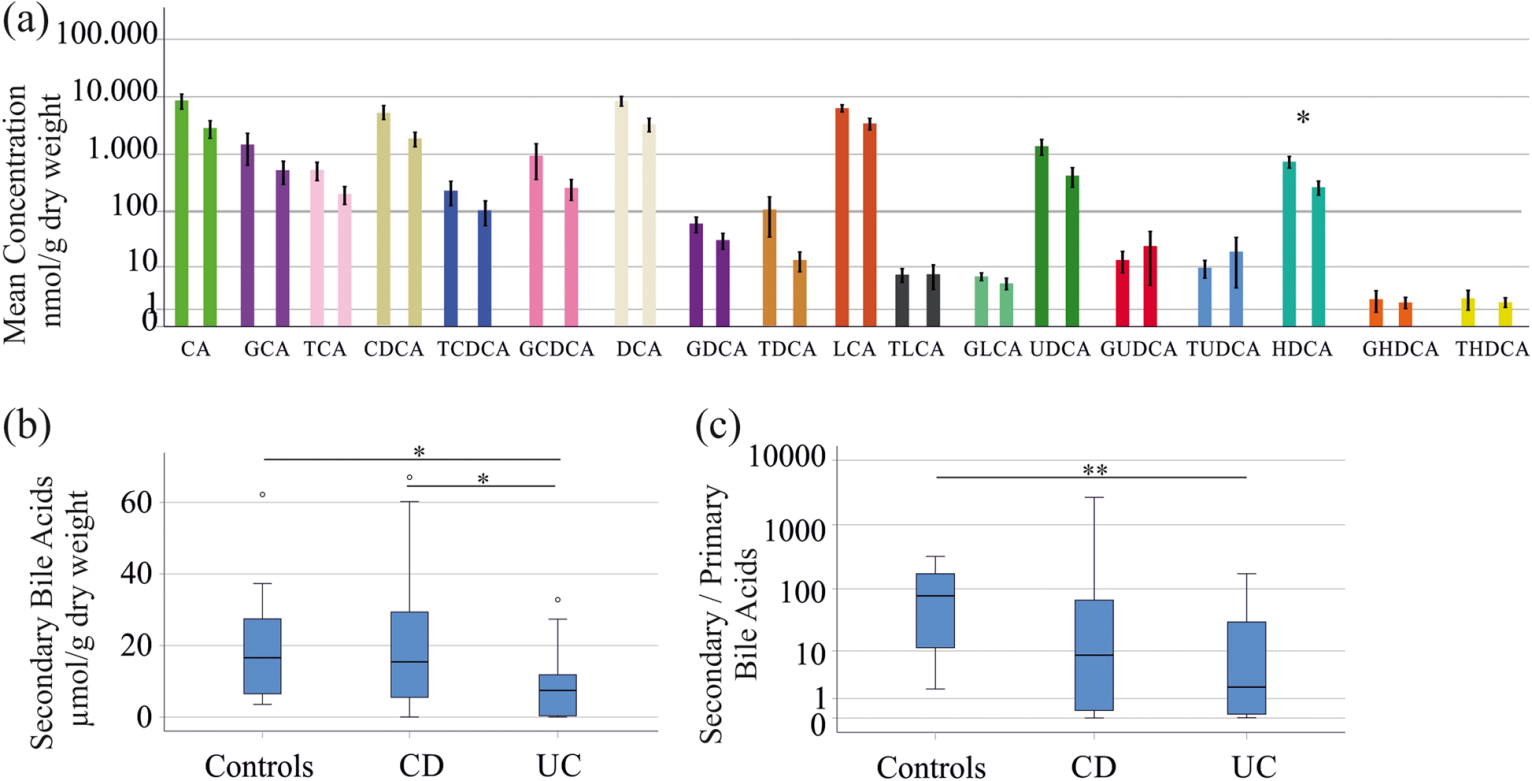
Fecal bile acids of CD and UC patients. **a** Fecal bile acids in stool of CD patients (left bars) and UC patients (right bars). Data are shown in a logarithmic scale to improve the visualization of different levels; **b** Secondary bile acids in stool of controls, CD and UC patients; **c** Ratio of secondary to primary bile acids in stool of controls, CD and UC patients. Data are shown in a logarithmic scale to improve the visualization of different levels. * *P* < 0.05, ** *P* < 0.01

There are distinct differences between CD and UC patients such as the location and depth of inflammation [7, 34], and therefore, both disease entities were analyzed separately and compared to healthy controls. CD patients had lower fecal levels of GHDCA (*P* = 0.008) compared to controls. UC patients exhibited lower levels of DCA (*P* = 0.036) and LCA (*P* = 0.01) in stool compared to healthy controls, with total secondary bile acid levels also being reduced (*P* = 0.036) (Table S2 and Fig. 2b).

It should be noted that CD and UC patients were comparable in age, BMI, CRP, and fecal calprotectin levels (Table 1). In CD %GHDCA (*P* = 0.007) was lower in contrast to controls. In UC %TCA (*P* = 0.035) was higher and %DCA (*P* = 0.008) was reduced in comparison to the controls. Accordingly, %primary bile acids of UC patients was higher (*P* = 0.004) and %secondary bile acids (*P* = 0.004) was lower in contrast to controls.

Fecal bile acid levels varied between CD and UC patients; CD patients had higher fecal HDCA (*P* = 0.033) and secondary bile acids (*P* = 0.045) compared to UC patients (Fig. 2a, b and Table S2). In CD %GHDCA (*P* = 0.02) was higher and %GCDCA (*P* = 0.04) was lower in comparison to UC.

Most studies agree that fecal primary bile acids increase, and secondary bile acids decrease in IBD [9, 11], and thus the ratio of secondary to primary bile acids was calculated. This ratio was low in UC patients in contrast to controls (*P* = 0.004) but did not significantly differ between CD patients and healthy controls (*P* = 0.080) (Fig. 2c).

### Correlations of fecal bile acid species with markers of inflammation

In the IBD cohort and the CD patients none of the bile acids correlated with serum CRP or fecal calprotectin (Table 2). In UC patients, DCA, TDCA, LCA, TLCA, UDCA and HDCA, and accordingly total levels of secondary bile acids, negatively correlated with CRP. GLCA, HDCA and secondary bile acids negatively correlated with fecal calprotectin in UC (Table 2).

**Table 2:** Spearman correlation coefficients for the correlations of fecal bile acids with CRP and fecal calprotectin in IBD, CD and UC. * *P* < 0.05, ** *P* < 0.01, *** *P* < 0.001, not significant n.s

	IBD	CD	UC	IBD	CD	UC
	Absolute Levels of Bile acids			Bile Acid Species/Total Bile Acid Level (%)		
**CRP**						
GCA	n.s.	n.s.	n.s.	n.s.	n.s.	0.643**
GCDCA	n.s.	n.s.	n.s.	n.s.	n.s.	0.807***
DCA	n.s.	n.s.	-0.741***	n.s.	n.s.	-0.607*
TDCA	n.s.	n.s.	-0.564*	n.s.	n.s.	n.s.
LCA	n.s.	n.s.	-0.790***	n.s.	n.s.	n.s.
TLCA	n.s.	n.s.	-0.605*	n.s.	n.s.	n.s.
UDCA	n.s.	n.s.	-0.667**	n.s.	n.s.	n.s.
HDCA	n.s.	n.s.	-0.627*	n.s.	n.s.	n.s.
Primary BA	n.s.	n.s	n.s.	n.s.	n.s.	n.s.
Secondary BA	n.s.	n.s	-0.834***	n.s.	n.s.	n.s.
**Fecal calprotectin**						
GCA	n.s.	n.s.	n.s.	n.s.	n.s.	0.619**
GCDCA	n.s.	n.s.	n.s.	0.355*	n.s.	0.609*
GLCA	n.s.	n.s.	-0.540*	n.s.	n.s.	n.s.
HDCA	n.s.	n.s.	-0.567*	n.s.	n.s.	-0.544*
GHDCA	n.s.	n.s.	n.s.	n.s.	n.s.	0.620**
Primary BA	n.s.	n.s.	n.s.	0.378*	n.s.	n.s.
Secondary BA	n.s.	n.s.	-0.553*	-0.378*	n.s.	n.s.

**Fig. 3:**
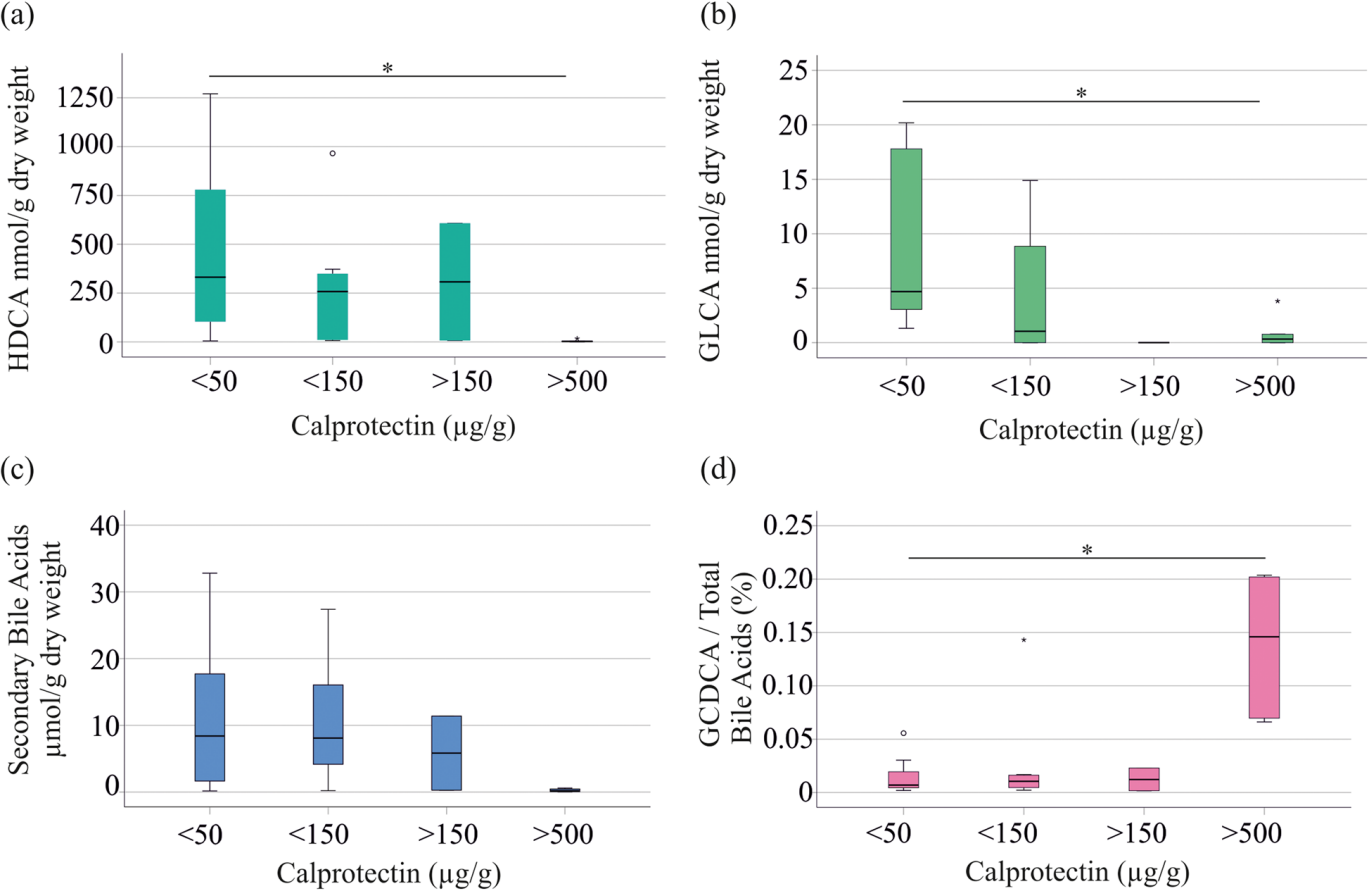
Relationship of fecal bile acids and fecal calprotectin. **a** HDCA levels in stool of UC patients with fecal calprotectin levels < 50 µg/g (8 patients), < 150 µg/g (8 patients), > 150 µg/g (2 patients) and > 500 µg/g (5 patients). Fecal calprotectin level of one patient was not documented; **b** GLCA in stool of UC patients categorized according to fecal calprotectin levels; **c** Secondary bile acids in stool of UC patients categorized according to fecal calprotectin levels; **d** %GCDCA in feces of UC patients in relation to fecal calprotectin levels. * *P* < 0.05

GLCA (*P* = 0.156), HDCA (*P* = 0.180), and total levels of secondary bile acids (*P* = 0.234) decreased with increasing fecal calprotectin in UC patients but these declines were not significant (Fig. 3a-c). However, the 8 UC patients with fecal calprotectin levels < 50 µg/ had higher fecal GLCA (*P* = 0.036), HDCA (*P* = 0.036) and a trend to increased levels of secondary bile acids (*P* = 0.066) compared to the 5 UC patients with calprotectin levels > 500 µg/g (Fig. 3a -c). In patients with CD, bile acid levels did not change with higher fecal calprotectin levels (data not shown).

**Fig. 4:**
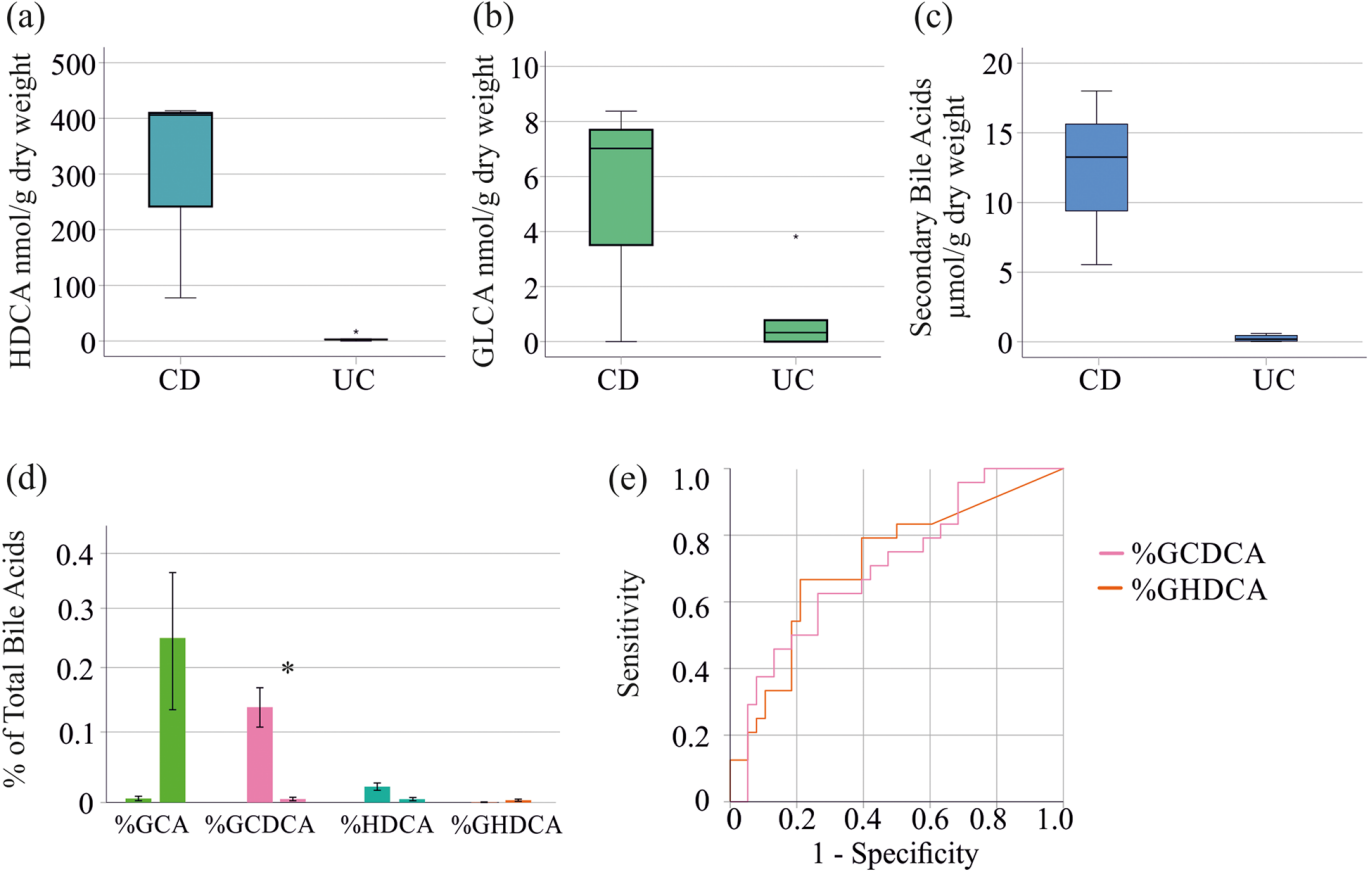
Bile acid levels of patients with fecal calprotectin levels > 500 µg/g and receiver operating characteristic curve. (**a**) HDCA; (**b**) GLCA and (**c**) Secondary bile acids in feces of CD and UC patients with fecal calprotectin levels > 500 µg/g; (**d**) %GCA, %GCDCA, %HDCA and %GHDCA of patients with CD (left bars) and UC (right bars), all with fecal calprotectin levels > 500 µg/g; (**e**) Receiver operating characteristic curve for diagnosis of UC including all IBD patients

HDCA, GLCA and secondary bile acids were much higher in feces of the 3 CD patients compared to the 5 UC patients, all with calprotectin levels > 500 µg/g. These differences were, however, not significant probably because of low patient number (Fig. 4a - c).

Relative bile acid levels did not exhibit a correlation with CRP and fecal calprotectin in CD patients (Table 2). In UC, the relative proportions of GCA and GCDCA showed positive associations with CRP and fecal calprotectin (Table 2). Negative correlations of %DCA and CRP, and %HDCA and fecal calprotectin were observed. However, the percentage of GHDCA exhibited a positive association with fecal calprotectin (Table 2). The 8 UC patients with fecal calprotectin levels < 50 µg/ had lower %GCDCA (*P* = 0.020) than the 5 UC patients with calprotectin levels > 500 µg/g (Fig. 3d). Percent GCA (*P* = 0.077), %HDCA (*P* = 0.474), and %GHDCA (*P* = 0.076) did not significantly differ among UC patients with low and high calprotectin levels.

The 5 UC patients with calprotectin levels > 500 µg/g had higher %GCA and %GHDCA, and lower %GCDCA and %HDCA compared to the 3 CD patients with calprotectin levels > 500 µg/g. Here, the difference of %GCDCA between CD and UC patients was significant (Fig. 4d).

In the IBD cohort, %GCDCA positively correlated with fecal calprotectin (Table 2). Relative levels of primary bile acids positively, and that of secondary bile acids negatively correlated with fecal calprotectin (Table 2).

A receiver operating characteristic curve (ROC) was used to evaluate the predictability of GLCA, HDCA and secondary bile acids for discrimination of UC from CD patients in the whole cohort (Fig. 4e). The area under the ROC curve (AUROC) was 0.422 for GLCA, 0.296 for HDCA, and 0.292 for secondary bile acids to diagnose UC. The AUROC of %GCA was 0.661, for %GCDCA was 0.722, for %HDCA was 0.423 and for %GHDCA was 0.712. An AUROC of 0.7 to 0.8 is considered acceptable for the discrimination of two cohorts [35], and ROC curves for %GHDCA and %GCDCA are shown in Fig. 4e. The Youden index method was used to define the optimal cut-points for UC diagnosis, which were 42.8 × 10^−6^% for GHDCA (specificity: 79%, sensitivity: 67%) and 0.008% for GCDCA (specificity: 74%, sensitivity: 63%).

### Relation of fecal bile acids with stool consistency

**Fig. 5:**
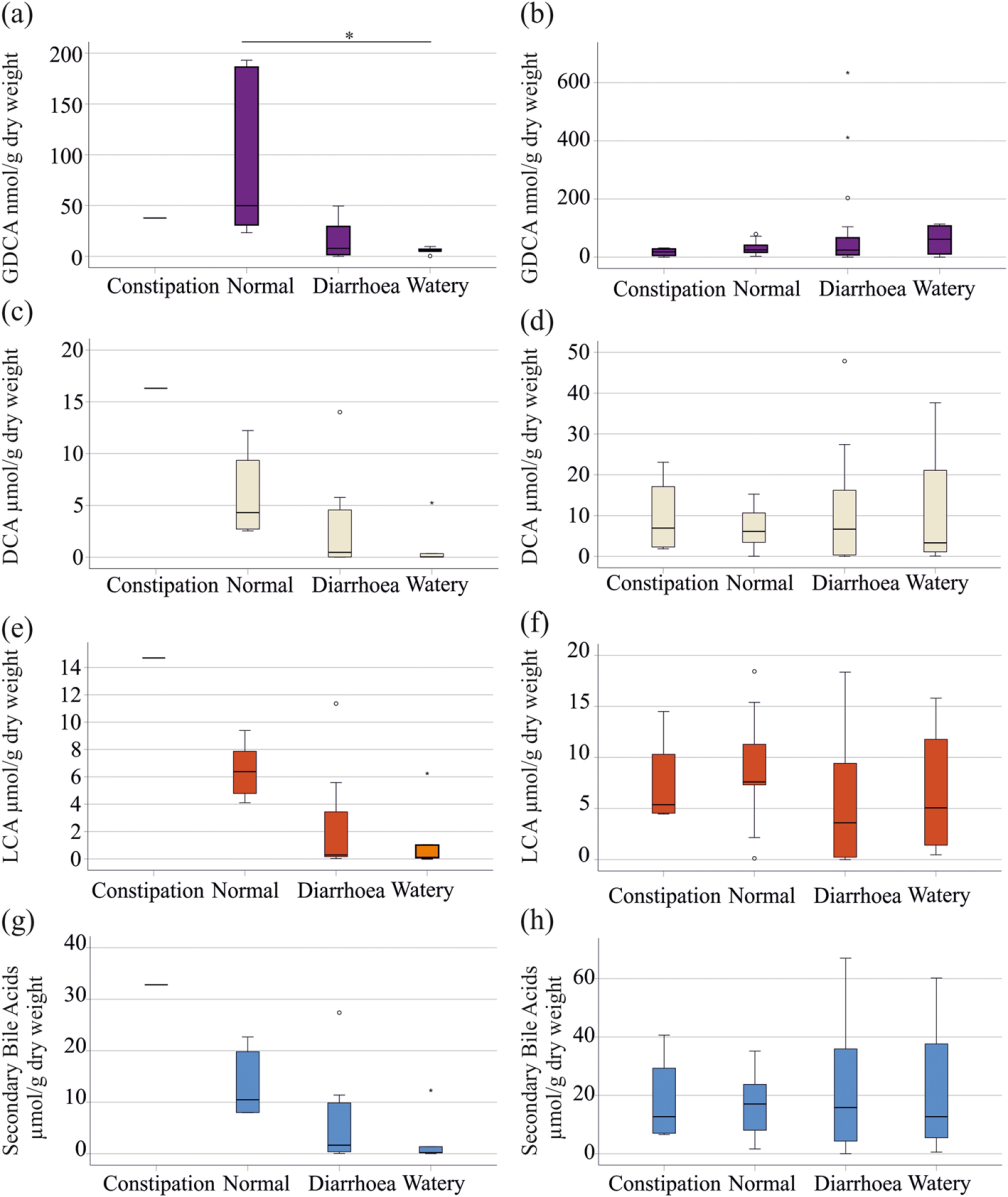
Relationship of fecal bile acids and stool consistency. **a** GDCA levels in stool of UC patients with constipation (1 patient), normal stool (6 patients), diarrhoea (12 patients) and watery stool (5 patients); **b** GDCA levels in stool of CD patients with constipation (4 patients), normal stool (9 patients), diarrhoea (21 patients) and watery stool (4 patients); (**c**) DCA in stool of UC patients described in a; **d** DCA in stool of CD patients described in b; **e** LCA in stool of UC patients described in a; **f** LCA in stool of CD patients described in b; **g** Secondary bile acids in stool of UC patients described in a; **h** Secondary bile acids in stool of CD patients described in b. * *P* < 0.05

Excess bile acids in the colon can cause diarrhoea but the association of fecal bile acids and stool consistency in IBD is unclear [13, 21, 36]. The type of the stool was documented by the patients using the Bristol stool chart, where type 1 and 2 are constipation, type 3 and 4 normal stool, type 5 and 6 diarrhoea and type 7 watery stool. There was a negative correlation of fecal DCA (r = -0.588, *P* = 0.015), GDCA (r = -0.682, *P* = 0.001) and LCA (r = -0.637, *P* = 0.005) with the Bristol stool scale in UC. Accordingly, there was a negative correlation of total secondary bile acids with the Bristol stool scale (r = -0.643, *P* = 0.004). Such correlations were not existent in CD patients (DCA: r = -0.035, *P* = 0.833, GDCA: r = 0.119, *P* = 0.280 and LCA: r = -0.180, *P* = 0.280). DCA, GDCA and LCA declined in stool of UC patients with higher water content but did not change in feces of CD patients (Fig. 5a - h). In UC, GDCA levels of patients with normal and watery stool differed significantly (Fig. 5a). There was also a trend for lower levels of secondary bile acids in UC but not CD patients with diarrhoea (Fig. 5g, h).

CRP and fecal calprotectin levels did not correlate with the Bristol stool chart in UC (r = 0.435, *P* = 0.259 and r = 0.404, *P* = 0.336, respectively).

### Association of fecal bile acids and medications

In IBD therapy, anti-TNF antibodies are commonly utilized [5]. In our cohort, 13 CD and 9 UC patients received this treatment. Total and relative levels of bile acids were similar in anti-TNF antibody treated and non-treated UC and CD patients (*P* > 0.05 for all).

**Fig. 6:**
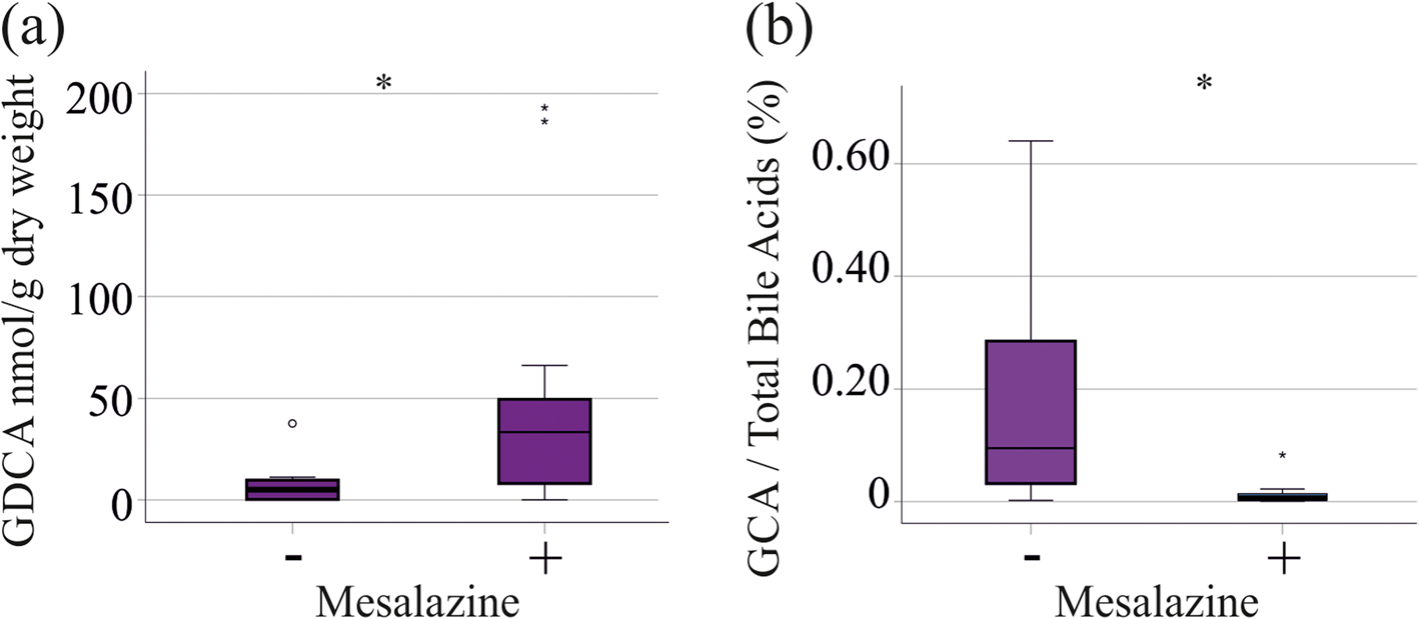
Associations of fecal bile acids with mesalazine therapy of UC patients. **a** GDCA levels of UC patients treated or not treated with mesalazine; **b** GCA levels relative to total bile acid concentration of UC patients treated or not treated with mesalazine. * *P* < 0.05

Corticosteroids were administered to 9 CD and 8 UC patients, with those treated exhibiting similar levels of bile acids as the non-treated patients (*P* > 0.05 for all). The bile acid profile was comparable between these two groups. Mesalazine (8 CD and 13 UC) was associated with higher GDCA (*P* = 0.046) and with lower %GCA (*P* = 0.032) in UC (Fig. 6a, b). UC patients treated and not-treated with this drug had similar levels of fecal calprotectin and CRP (*P* = 0.111 and *P* = 0.981, respectively). Anti-interleukin 12/23 antibody therapy (12 CD and 6 UC), and azathioprine (4 CD and 2 UC) treatments were not associated with changes of fecal bile acid levels (data not shown).

## Discussion

The present study corroborated prior research, indicating that primary fecal bile acid levels increase, while secondary bile acid levels decrease in IBD patients compared to healthy controls [9, 11]. Notably, fecal bile acid levels demonstrated associations with biomarkers for IBD severity in UC patients, potentially enhancing diagnostic value.

Feces is heterogeneous, and mainly consists of indigestible solid material and water [37]. To compare stool lipid levels among different individuals, an accurate normalization is needed. Sample wet weight, stool dry weight, and fecal protein concentration have been used for normalization of bile acid levels, and bile acid concentration of individual samples greatly differed concerning the type of normalization [15].

Patients with IBD may experience loose, watery stool [37], and thus, bile acid levels were normalized to stool dry weight to account for variations in water content. Calculating the ratios of individual bile acid species to total levels provides a viable method for comparing different cohorts, an approach also utilized in our study.

Fecal bile acids of patients with IBD have been analyzed by several research groups [2, 12, 13, 16, 25]. Higher fecal levels of primary together with lower levels of secondary bile acids in IBD appeared to be consistent findings across the studies [2].

In our cohort, lower fecal levels of secondary bile acids, and accordingly a reduced ratio of secondary to primary bile acids, in comparison to healthy controls was identified in UC but not CD patients. Accordingly, healthy controls as well as CD patients had higher levels of fecal secondary bile acids compared to UC with no differences between the first two groups.

Our study also showed that fecal bile acids may be of additional diagnostic value in IBD. Negative correlations of the secondary bile acids DCA, TDCA, LCA, TLCA, UDCA and HDCA, and total levels of secondary bile acids with CRP were observed in UC. GLCA and HDCA, and total levels of secondary bile acids negatively correlated with fecal calprotectin in UC. The notable decrease of HDCA and GLCA, and the increase of GCDCA relative to total bile acid levels in feces of UC patients with high fecal calprotectin, compared to those with low fecal calprotectin, was distinctly observed in UC. This effect was not present in CD, underscoring a clinically relevant characteristic potentially specific to UC.

Fecal levels of GLCA and HDCA as well as %GCDCA and %HDCA were much lower in active UC compared to active CD. Additionally, patients with active UC had higher %GCA and %GHDCA in comparison to patients with active CD. The group of patients with active disease included only 3 CD and 5 UC patients, and these differences were mostly not significant.

An AUROC of 0.7 to 0.8 is considered acceptable for the discrimination of two cohorts [35, 38, 39], and this applied to the relative levels of GHDCA and GCDCA. Percent GHDCA had a specificity of 79% and a sensitivity of 67% and %GCDCA a specificity of 74% and a sensitivity of 63% for diagnosis of UC. A non-invasive test for diagnosis of UC versus CD has been described and anti-neutrophil cytoplasmic antibodies had a specificity of 89% and a sensitivity of 55% [40]. Future studies have to evaluate the clinical validity of these biomarkers to distinguish UC from CD patients.

Present and previous [25] observations indicate that higher levels of primary bile acids and lower levels of secondary bile acids are associated with inflammation in UC. The relative abundance of the secondary bile acid GHDCA was, however, positively correlated with fecal calprotectin in UC suggesting that not only the absolute levels of bile acids but in addition the bile acid pool composition are related with UC severity.

Bile acid deconjugation and formation of secondary bile acids by bacteria takes place in the colon [13]. UC is characterized by chronic mucosal inflammation extending from the rectum to the more proximal colon [41] whereas transmural inflammation of the intestine in CD can affect any part of the gastrointestinal tract [6]. While further investigations are needed to determine whether the distinct localization of inflammatory processes contributes to the varied bile acid profiles between CD and UC, the fecal bile acid composition holds potential diagnostic value in differentiating between the two disease entities, presenting therapeutic relevance [42].

Diarrhoea is common in IBD and fecal bile acids may contribute to watery stool [21]. DCA, GDCA and LCA levels of UC patients with diarrhoea were, however, low in contrast to UC patients with normal stools. Again, such associations were not observed in CD. Stool consistency evaluated by the Bristol stool chart was not related to CRP and fecal calprotectin levels, and thus was not associated with disease severity. Until now, the pathogenesis of IBD diarrhoea has not been well described, and the role of bile acids is still debated [21]. Future studies have to clarify the relation of secondary bile acid homeostasis and diarrhoea in UC patients.

Corticosteroids are widely used anti-inflammatory drugs, which may produce undesirable effects such as lipid abnormalities [43]. Prednisolone treatment of mice increased ileal bile acid absorption, elevated plasma bile acid levels and reduced their fecal levels [44, 45]. In our IBD cohort, patients receiving corticosteroids did not exhibit reduced fecal bile acid levels. Anti-interleukin 12/23 antibody therapy and azathioprine treatments were not associated with changes in fecal bile acid levels. Notably, mesalazine was related to higher fecal GDCA levels, and a decline of %GCA in UC. Mesalazine is a well-known drug for the treatment of IBD patients, and is used in mild-to-moderate illnesses [46]. CRP as well as fecal calprotectin levels of patients treated with mesalazine in comparison to patients treated with different medications were similar indicating that the rise of GDCA levels and the decline of %GCA may be a specific effect of this drug. Because this study cohort was quite small, this finding awaits confirmation.

### Strengths and limitations

To our knowledge, this represents the first study exploring associations between fecal calprotectin and bile acid levels, benefiting from the strength of a relatively large cohort that facilitates the comparison between CD and UC patients. A limitation includes collecting only a single fecal sample per patient at varied times throughout the day. While the study is descriptive and does not provide explanations for observed differences between UC and CD patients, it lays a foundational framework for subsequent investigations in this field.

## Conclusions

Fecal bile acid composition is altered in UC and may serve as a useful parameter for distinguishing between CD and UC, enhancing differential diagnosis, which can still be a challenge. Relative levels of GCDCA and GHDCA may be used in conjunction with other biomarkers to distinguish UC from CD patients. GLCA and HDCA as well as total levels of secondary bile acids are low in active UC, and may offer additional value for monitoring disease severity. This study also observed associations of fecal DCA, GDCA and LCA with diarrhoea in UC patients. The pathophysiological role and diagnostic value of secondary bile acid species, however, should be further evaluated in larger cohorts.

### Supplementary Information

**Additional file 1: Table S1.** Bile acid (BA) species levels in stool of controls and IBD patients. All bile acid concentrations are given in nmol/g dry weight. Significant different median levels are in bold. * *P* < 0.05. **Table S2.** Bile acid species levels in stool of controls, CD and UC patients. All bile acid concentrations are given in nmol/g dry weight. Significant different median levels are in bold. * *P* < 0.05 for comparison between controls and CD or UC patients. ^§^*P* < 0.05, ^§§^*P* < 0.01 for comparison of CD and UC.

## Data Availability

Original data can be obtained by the corresponding author on request.

## Bile acid species

CA: Cholic acid
GCA: Glycine conjugated CA
TCA: Taurine conjugated CA
CDCA: Chenodeoxycholic acid
GCDCA: Glycine conjugated CDCA
TCDCA: Taurine conjugated CDCA
DCA: Deoxycholic acid
GDCA: Glycine conjugated DCA
TDCA: Taurine conjugated DCA
LCA: Lithocholic acid
GLCA: Glycine conjugated LCA
TLCA: Taurine conjugated LCA
UDCA: Ursodeoxycholic acid
GUDCA: Glycine conjugated UDCA
TUDCA: Taurine conjugated UDCA
HDCA: Hyodeoxycholic acid
GHDCA: Glycine conjugated HDCA
THDCA: Taurine conjugated HDCA

## Further abbreviations

CD: Crohn´s disease
UC: Ulcerative colitis
