# Supplementary material for: Altered fecal bile acid composition in active ulcerative colitis
Source: Lipids Health Dis. 2023 Nov 18;22:199. doi: 10.1186/s12944-023-01971-4 (PMC10656844; doi:10.1186/s12944-023-01971-4)
Supplement: Supplementary file 1 — Additional file 1: Table S1. Bile acid (BA) species levels in stool of controls and IBD patients. All bile acid concentrations are given in nmol/g dry weight. Significant different median levels are in bold. * P < 0.05. Table S2. Bile acid species levels in stool of controls, CD and UC patients. All bile acid concentrations are given in nmol/g dry weight. Significant different median levels are in bold. * P < 0.05 for comparison between controls and CD or UC patients. §P < 0.05, §§P < 0.01 for comparison of CD and UC. [file 12944_2023_1971_MOESM1_ESM.docx]

Altered Fecal Bile Acid Composition in Active Ulcerative Colitis

Stefanie Sommersberger ^1^, Stefan Gunawan ^1^, Tanja Elger ^1^, Tanja Fererberger ^1^, Johanna Loibl ^1^, Muriel Huss ^1^, Arne Kandulski ^1^, Sabrina Krautbauer ^2^, Martina Müller ^1^, Gerhard Liebisch ^2^, Christa Buechler ^‡^ ^1^ * and Hauke Christian Tews ^‡^ ^1^

Table S1: Bile acid (BA) species levels in stool of controls and IBD patients. All bile acid concentrations are given in nmol/g dry weight. Significant different median levels are in bold. * *P* < 0.05.

|  | Controls | | | IBD | | | *P* |
| --- | --- | --- | --- | --- | --- | --- | --- |
|  | Median | Min | Max | Median | Min | Max |  |
| CA | **0.00** | **0.00** | **4587.26** | **616.96** | **0.00** | **73855.29** | * |
| GCA | 64.90 | 7.54 | 924.35 | 94.18 | 0.97 | 33064.58 |  |
| TCA | 3.53 | 0.00 | 561.34 | 27.08 | 0.00 | 5398.21 |  |
| CDCA | 24.26 | 0.00 | 4399.02 | 665.53 | 0.00 | 38667.17 |  |
| GCDCA | 67.83 | 26.23 | 632.57 | 78.80 | 0.00 | 23116.35 |  |
| TCDCA | 0.00 | 0.00 | 81.79 | 0.00 | 0.00 | 2838.87 |  |
| DCA | 7762.43 | 710.46 | 34003.68 | 3700.87 | 0.00 | 47847.41 |  |
| GDCA | 29.29 | 4.23 | 387.41 | 21.91 | 0.00 | 634.22 |  |
| TDCA | 1.31 | 0.00 | 202.45 | 2.46 | 0.00 | 2802.98 |  |
| LCA | 8401.19 | 2112.54 | 23499.31 | 4560.40 | 0.53 | 18423.33 |  |
| GLCA | 5.06 | 0.00 | 8.59 | 3.30 | 0.00 | 26.91 |  |
| TLCA | 0.32 | 0.00 | 8.90 | 1.45 | 0.00 | 86.29 |  |
| UDCA | 123.02 | 0.00 | 2441.89 | 221.13 | 0.00 | 15442.02 |  |
| GUDCA | 0.00 | 0.00 | 58.39 | 0.00 | 0.00 | 510.89 |  |
| TUDCA | 0.00 | 0.00 | 9.93 | 1.28 | 0.00 | 384.18 |  |
| HDCA | 387.73 | 48.84 | 2220.27 | 273.36 | 0.00 | 6008.50 |  |
| GHDCA | **1.30** | **0.00** | **13.42** | **0.54** | **0.00** | **47.15** | ***** |
| THDCA | 0.87 | 0.00 | 12.41 | 0.47 | 0.00 | 45.20 |  |
|  |  |  |  |  |  |  |  |
| Primary BA | 186.03 | 64.73 | 9240.26 | 1736.50 | 4.99 | 99587.07 |  |
| Secondary BA | 16580.29 | 3564.03 | 62205.51 | 10054.14 | 26.18 | 67022.17 |  |
| Total BA | 18146.43 | 3700.36 | 71445.77 | 18378.51 | 172.81 | 116770.94 |  |

Table S2: Bile acid species levels in stool of controls, CD and UC patients. All bile acid concentrations are given in nmol/g dry weight. Significant different median levels are in bold. * *P* < 0.05 for comparison between controls and CD or UC patients. ^§^ *P* < 0.05, ^§§^ *P* < 0.01 for comparison of CD and UC.

|  | Controls | | | CD | | | UC | | |
| --- | --- | --- | --- | --- | --- | --- | --- | --- | --- |
|  | Median | Min | Max | Median | Min | Max | Median | Min | Max |
| CA | 0.00 | 0.00 | 4587.26 | 638.35 | 0.00 | 73855.29 | 505.01 | 0.00 | 21340.37 |
| GCA | 64.90 | 7.54 | 924.35 | 86.93 | 4.99 | 33064.58 | 98.24 | 0.97 | 5167.28 |
| TCA | 3.53 | 0.00 | 561.34 | 23.95 | 0.00 | 5398.21 | 30.04 | 0.00 | 1307.02 |
| CDCA | 24.26 | 0.00 | 4399.02 | 785.88 | 0.00 | 38667.17 | 446.54 | 0.00 | 11606.08 |
| GCDCA | 67.83 | 26.23 | 632.57 | 83.38 | 0.00 | 23116.35 | 74.79 | 21.88 | 2591.74 |
| TCDCA | 0.00 | 0.00 | 81.79 | 0.00 | 0.00 | 2838.87 | 0.00 | 0.00 | 851.66 |
| DCA | **7762.43*** | **710.46** | **34003.68** | 5310.05 | 0.00 | 47847.41 | **1691.20*** | **0.00** | **16319.21** |
| GDCA | 29.29 | 4.23 | 387.41 | 24.28 | 0.00 | 634.22 | 10.45 | 0.00 | 193.10 |
| TDCA | 1.31 | 0.00 | 202.45 | 2.19 | 0.00 | 2802.98 | 2.62 | 0.00 | 109.40 |
| LCA | **8401.19*** | **2112.54** | **23499.31** | 5198.78 | 0.53 | 18423.33 | **2239.87*** | **2.84** | **14691.95** |
| GLCA | 5.06 | 0.00 | 8.59 | 4.85 | 0.00 | 26.91 | 2.12 | 0.00 | 20.18 |
| TLCA | 0.32 | 0.00 | 8.90 | 1.71 | 0.00 | 71.15 | 1.45 | 0.00 | 86.29 |
| UDCA | 123.02 | 0.00 | 2441.89 | 430.43 | 0.00 | 15442.02 | 55.97 | 0.00 | 3313.81 |
| GUDCA | 0.00 | 0.00 | 58.39 | 0.00 | 0.00 | 177.14 | 0.00 | 0.00 | 510.89 |
| TUDCA | 0.00 | 0.00 | 9.93 | 0.45 | 0.00 | 110.27 | 2.06 | 0.00 | 384.18 |
| HDCA | 387.73 | 48.84 | 2220.27 | **372.27^§^** | **5.72** | **6008.50** | **133.37^§^** | **0.00** | **1270.16** |
| GHDCA | **1.30**** | **0.00** | **13.42** | **0.39**** | **0.00** | **47.15** | 0.70 | 0.00 | 12.91 |
| THDCA | 0.87 | 0.00 | 12.41 | 0.21 | 0.00 | 45.20 | 0.70 | 0.00 | 11.79 |
|  |  |  |  |  |  |  |  |  |  |
| Primary BA | 186.03 | 64.73 | 9240.26 | 2613.21 | 4.99 | 99587.07 | 1446.85 | 48.38 | 33220.15 |
| Secondary BA | **16580.29*** | **3564.03** | **62205.51** | **15432.58^§§^** | **36.52** | **67022.17** | **7461.46^*,§§^** | **26.18** | **32808.67** |
| Total BA | 18146.43 | 3700.36 | 71445.77 | 30513.98^§^ | 3202.71 | 116770.94 | 10246.77^§^ | 172.81 | 44603.09 |
